# Vertical distribution of megafauna on the Bering Sea slope based on ROV survey

**DOI:** 10.7717/peerj.8628

**Published:** 2020-03-02

**Authors:** Elena Rybakova, Sergey Galkin, Andrey Gebruk, Nadezhda Sanamyan, Alexander Martynov

**Affiliations:** 1 Shirshov Institute of Oceanology, Russian Academy of Sciences, Moscow, Russia; 2 Kamchatka Branch of Pacific Geographical Institute, Far-Eastern Branch of the Russian Academy of Sciences, Petropavlovsk-Kamchatsky, Russia; 3 Zoological Museum, Moscow State University, Moscow, Russia

**Keywords:** Bering Sea, ROV survey, Vertical distribution, Vertical zonation, Megafauna, Volcanologists Massif, Komandorsky Basin, Biodiversity

## Abstract

Video surveys were carried out during the 75th cruise of the RV *Akademik M.A. Lavrentyev* (June 2016) along the northern slope of the Volcanologists Massif, in the south-western Bering Sea. The seafloor was explored using the ROV *Comanche 18*. Seven dives were performed in the depth range from 4,278 m to 349 m. Overall, about 180 species of megafauna were recognised. Fifteen types of megafauna communities corresponding to certain depth ranges were distinguished based on the most abundant taxa. Dominance changed with depth in the following order: the holothurian *Kolga kamchatica* at the maximum depth (4,277–4,278 m); the holothurian *Scotoplanes kurilensis* at 3,610–2,790 m; the ophiuroid *Ophiura bathybia* at 3,030–2,910 m; benthic shrimps of the family Crangonidae at 2,910–2,290 m; the holothurian *Paelopatides solea* at 2,650–2,290 m; benthic jellyfish from the family Rhopalonematidae at 2,470–2,130 m; the enteropneust Torquaratoridae at 2,290–1,830 m; the holothurian *Synallactes chuni* and the ophiuroid of the genera *Ophiura* and *Ophiocantha* at 1,830–1,750 m. At depths 1,750–720 m most of the megafauna was associated with live or dead colonies of the sponge *Farrea* spp. Depths 720–390 m were dominated by the coral *Heteropolypus ritteri* and/or *Corallimorphus pilatus.* At 390–350 m depth, the shallowest depth range, the dominant taxon was the zoantharian *Epizoanthus* sp. Soft sediment megafauna communities dominated by torquaratorid enteropneusts to our knowledge have not been observed before in the deep-sea, the same as communities with a dominance of benthopelagic rhopalonematid jellyfish. The depths of the largest community changes, or the largest turnover of dominant species, were revealed at ∼2,790 m between the bathyal and abyssal zones and ∼1,750 m and ∼720 m within the bathyal zone.

## Introduction

Depth plays a crucial role in the distribution of species and communities in the World Ocean. It was shown that species generally occupy discrete depth ranges and progressively replace each other from the continental shelf to the abyssal depths (Carney, Haedrich & Rowe, 1983). Distinct bathymetric faunal zones have been identified for many marine taxa (Mironov, 1986; Billett, 1991; Cartes & Sarda, 1993) and for marine fauna in general (Rowe & Menzies, 1969; Menzies, George & Rowe, 1973; Haedrich, Rowe & Polloni, 1975). Very few studies have examined the depth-related distribution of deep-sea species within their depth ranges to understand not only critical depths of faunal change but to identify more subtle changes that are related to the abundance of species that can provide a better overall picture of faunal zonation. For example, Howell, Billett & Tyler (2002) showed that asteroid species have very narrow centres of distribution in which they are abundant, despite much wider total adult depth ranges. Depth-related faunistic boundaries occur at many locations worldwide indicating that important controlling factors are present at these depths and that they may occur globally. Causes of the vertical zonation are related to the biology and physiology of the marine organisms and controlled by change with depth of some pivotal environmental parameters such as pressure, light, temperature and food availability (Menzies, George & Rowe, 1973; Rex, 1976; Carney, 2005; Rex & Etter, 2010). Generally, the causes of change in species composition with depth are complex, and several factors might interact, leading to the certain patterns observed in the each specific area (Soltwedel et al., 2009).

Benthic megafauna may be defined as marine animals exceeding 0.5–1 cm in size and recognizable on seafloor images (Grassle et al., 1975; Rex, 1981). Information on abundance and distribution of the megafauna is important for deep-sea benthic ecology. Megafaunal organisms play an important role in benthic ecosystems through active recycling of sediment organic matter, bioturbation and food web linkages.

The number of megafaunal studies has increased with the growing use of deep-sea video and imagery for ecological purposes.

Investigation of the Bering Sea began in the 18th century by G.W. Steller during the V. Bering expedition (1,737–1,742). Various species from the Bering Sea were studied in the late 19th and early 20th century. The first quantitative results of the benthos distribution were obtained in 1,932–1,933 by K. Derugin (Ushakov & Kusakin, 1978). Further considerable contributions were made by expeditions of the RV *Vityaz* in 1,950–1,952 to the western part of the Bering Sea and by the expeditions of a number of research vessels in 1,958–1,960 to the eastern part of the Bering Sea (Ushakov, 1952; Vinogradova, 1954; Neyman, 1960; Neiman, 1963; Zenkevitch, 1963; Filatova & Barsanova, 1964; Kuznetzov, 1964; Semenov, 1964; Vinogradov & Neyman, 1965). Qualitative and quantitative studies of the distribution of the benthos and trophic structure were described mainly on the shelf and upper slope (Rowland, 1973; Stoker, 1973; Alton, 1974; Haflinger, 1978; Stoker, 1978; Jewett & Feder, 1981) while surveys of the Western (Komandorsky) and Central (Aleutian) Basins were extremely limited. Impact of climate change on the Bering Sea ecosystems is being actively studied; however, these investigations are restricted only to some economically important species and do not take into account benthic communities as a whole (Hamazaki et al., 2005; Grebmeier et al., 2006; Stevenson & Lauth, 2012). Available data on the deep basins of the Bering Sea were obtained using trawls and grabs. Image and video surveys of the deep-sea megafauna communities were not conducted before, with the exception of dives in the submersible *Mir* in 1990 to the Piip’s Volcano. Based on these dives, vertical zonation of benthic fauna on the upper part of the northern and southern slope of the Volcanologists Massif was described (Galkin & Sagalevich, 2012). It was mentioned about 30 species of megafauna in the depth range from 450 to 900 m and the vertical zonation in fauna distribution was revealed. Dominant taxa changed with depth with hexactinellid sponges in the lower part replaced by corals and unidentified small cnidarians in the upper part.

The Bering Sea is separated from the North Pacific Ocean by the Aleutian-Komandor Island systems. The channels between the islands often have a great depth, sometimes exceeding 4,000 m, permitting almost unrestricted exchange between the abyssal zone of the Bering Sea and the Pacific Ocean (Stoker, 1978). As a result, most of the deep-sea species of the Bering Sea are in common with the Pacific Ocean (Filatova & Barsanova, 1964). By contrast, the exchange with the Chukchi Sea and the Arctic Ocean is limited to the Bering Strait, less than 50 m deep. The biomass of benthos in the Komandorsky Basin is very high—up to 32 g/m^2^, average 14 g/m^2^ (Belyev, 1960). It is explained by favourable nutritional conditions due to proximity to the slope, abundant flora and fauna of the sublittoral zone and currents carrying organic-rich water from bays and mixing with the deep layers. For comparison, in the Central Basin biomass is lower due to isolation from the shelf (average 6 g/m^2^) (Filatova & Barsanova, 1964).

At least seven physical factors may influence the qualitative and quantitative distribution of the Bering Sea benthic fauna. These factors include sediment particle size, bottom temperature, salinity, depth, sedimentation rates, circulation intensity, and suspended particle content in the near-bottom water layer (Kuznetzov, 1964; Stoker, 1978). Usually species dominating faunal assemblages belong to several trophic groups (Neyman, 1960; Filatova & Barsanova, 1964; Stoker, 1973); however, one certain trophic type can prevail. The distribution of different trophic types depends on topography, distribution of organic carbon in the sediment, mobility of bottom waters and distribution of erosion and sedimented zones at the seafloor (Neiman, 1963; Rex, 1981; Gage & Tyler, 1991; Sokolova, 2000). For example, sessile suspension feeders are dominated in the Bering Sea at areas with high speed of currents, at areas with high concentrations of suspension particles in near bottom layers of water and on hard substrate (Kuznetzov, 1964). Deposit-feeders are dominated at areas with soft sediment enriched with organic carbon (Gage & Tyler, 1991).

The present study is the first detailed investigation on the megafauna communities of the slope of the Volcanologists Massif and adjacent area of the Komandorsky Basin based on video survey. Our main aim is to clarify patterns of the vertical distribution of megafauna communities on the slope of the Volcanologists Massif based on their composition and structure.

## Material & Methods

### Study area and video surveys

**Figure 1:**
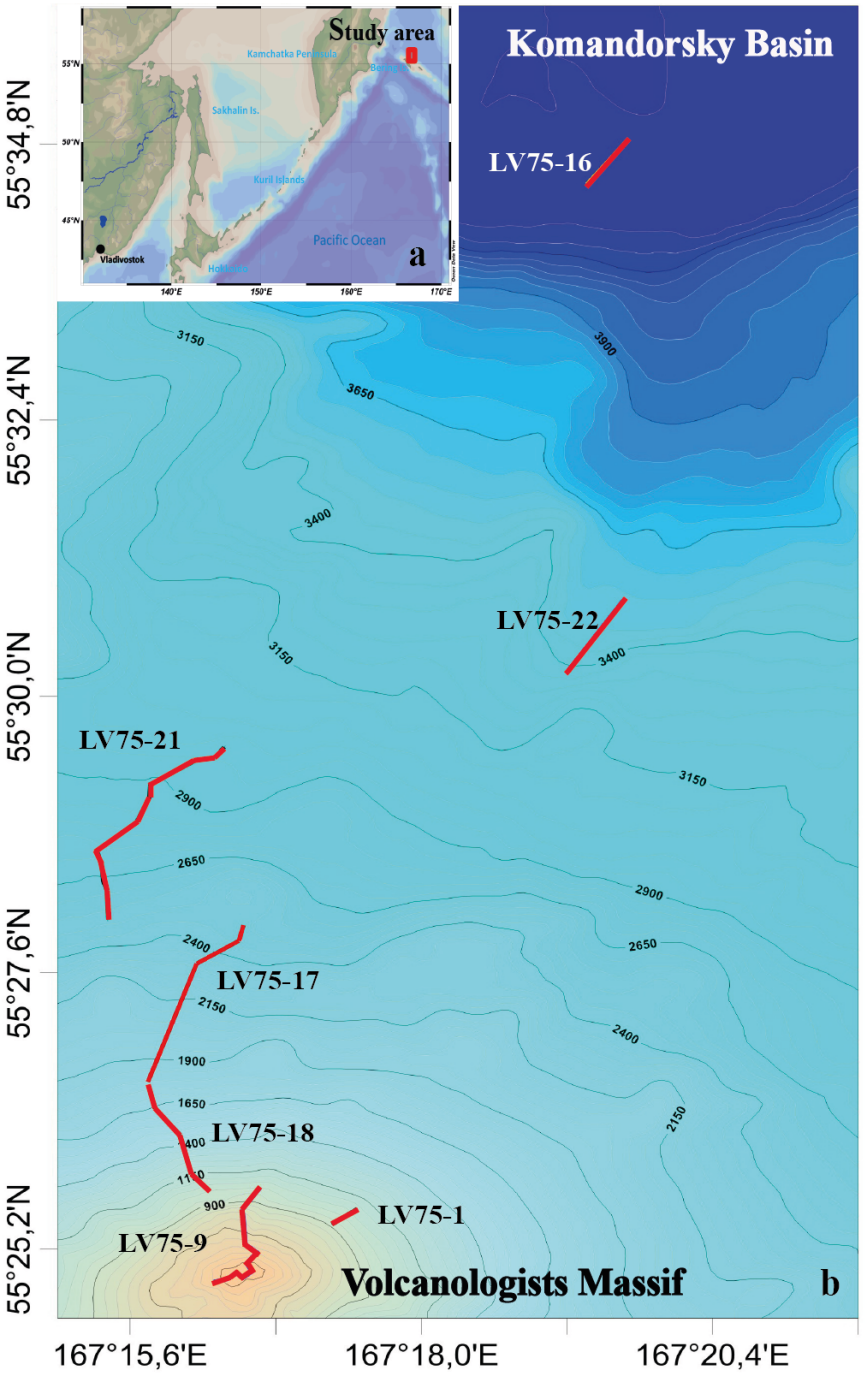
The tracks of the 7 ROV *Comanche 18* dives on the northern slope of the Volcanologists Massif, in the south-west Bering Sea (75th cruise of RV *Akademik M.A. Lavrentyev*, June 2016). Five of the dives are broken up further into quantitative transects as explained in Table 1.

Video surveys were carried out during the 75th cruise of the RV *Akademik M.A. Lavrentyev* (June 2016) in the northern slope of the Volcanologists Massif, the south-west Bering Sea (Fig. 1). The seafloor was explored using the ROV *Comanche 18* (Galkin & Ivin, 2019). Seven dives were performed in the depth range from 4,278 m to 349 m. For technical reasons, dives tracks were directed upwards along the slope. No specific permissions were required for the investigated location because it is the territorial waters of the Russian Federation and it is not the protected area.

Visual observations were conducted in a continuous mode, total duration is 40 hrs 44 min. Observations were accompanied by video recording and sampling of individual organisms by ROV manipulator. The field studies did not involve endangered or protected species. The ROV was equipped with the Kongsberg Underwater HDTV colour camera OE14-502 and with a ten-centimetre laser scale. A sonardyne USBL Fusion underwater navigation system was used. In total 22 hrs 29 min of video footage were analysed. Dive details are given in **Table 1**.

**Table 1:** Details of ROV *Comanche 18* dives and quantitative video transects.

**Dive (#)**	**Date**	**Time on the bottom**			**Coordinates (observation)**				**Depth**		**Quantitative transect (#)**	**Duration (min:sec)**	**Estimated length (m)**	**Mean depth (m)**
		**Start**	**End**	**Total (hour:min)**	**Start**		**End**		**Start**	**End**				
					**Latitude (N)**	**Longitude (E)**	**Latitude (N)**	**Longitude (E)**						
16	26.06.2016	17:57	21:08	3:11	55.5774	167.3258	55.5760	167.3249	4,278	4,277	1	3:07	144	4,277
											2	3:40	169	4,277
											3	2:24	111	4,277
											4	1:36	74	4,277
22	02.07.2016	10:17	14:20	4:03	55.5143	167.3272	55.5039	167.3195	3,610	3,451	5	3:02	140	3,603
											6	4:10	192	3,587
											7	4:13	195	3,583
											8	8:15	381	3,494
21	01.07.2016	10:34	19:32	8:58	55.4934	167.2726	55.4683	167.2537	3,023	2,502	9	8:00	370	2,983
											10	5:00	232	3,023
											11	5:00	232	2,954
											12	5:00	232	2,960
											13	5:00	232	2,890
											14	5:00	232	2,890
											15	5:05	235	2,849
											16	3:40	169	2,847
											17	4:40	216	2,847
											18	5:00	232	2,790
											19	5:00	232	2,732
											20	5:00	232	2,626
											21	5:00	232	2,555
											22	3:00	139	2,502
											23	5:00	232	2,502
17	27.06.2016	14:40	22:51	8:11	55.4665	167.2756	55.4438	167.2629	2,487	1,800	24	5:00	232	2,461
											25	5:00	232	2,441
											26	5:00	232	2,380
											27	2:40	123	2,218
											28	4:00	185	1,914
											29	3:25	158	1,850
18	28.06.2016	9:45	15:59	6:14	55.4438	167.2628	55.4285	167.2699	1,806	992	30	6:00	277	1,785
											31	2:40	123	1,750
											32	2:30	115	1,650
											33	2:30	115	1,600
1	11.09.2016	10:28	12:13	1:45	55.4242	167.2922	55.4251	167.2905	1,077	1,055	–	–		–
9	19.06.2016	10:38	19:00	8:22	55.4289	167.2779	55.4299	167.2773	1,013	349	–	–		–

The present investigation was focused on soft sediment epibenthic megafauna. To estimate the relative abundance of the soft sediment megafauna, a series of video transects were performed at depths 4,278–1,370 m. Transects for quantitative analysis were selected from five dives: 16, 22, 21, 17 and 18. Sediment cover on these transects exceeded 70% of the total seafloor square. The number of transects selected for the analysis from each dive depended on the quality of video records. The quality in turn was subject to conditions of dive (speed and altitude) influenced by the seafloor topography, currents and technical aspects. Transects were selected if they complied with the following characteristics: camera was looking almost vertically down, average speed was about 0.5 knots, altitude was approximately 1.5 m, there were no sediment clouds and illumination was satisfactory (**Table 1**). Altogether 33 quantitative transects were analysed with total time of 2 hrs 25 min. The time of individual transects varied from 1.5 min to 8 min (about 5 min in most cases). The fauna of the hard substrate on all dives was described only qualitatively due to difficulties in adequate identification to a low taxonomic level and counting of animals on hard substrate with complex topology.

On two dives (1 and 9) at depths 1,370–350 m, quantitative transects could not be selected owing to the predominance of hard substrata and extremely rough topography. However, records from these transects were used for qualitative analysis of benthic communities.

### Video analysis

Video records were analysed on land using the QuickTime software*.* Visible megafauna was registered and taxonomic experts provided identification of the observed organisms to the lowest possible taxonomic level. For identification of some species, specimens were collected using the ROV manipulator. Complete lists of taxa identified on video and in collected material are given in Table S1. Images of identified taxa on video are given in Data S1. Association with hard or soft substrate was noted for all identified species. Abundance of all identified species on hard and soft substrate was evaluated visually using four-point scale based on video (Table S1).

All specimens of each visible soft sediment megafauna species on quantitative transects were counted (Table S2). The following taxa/organisms were excluded from the statistical analysis because they could not be adequately identified and/or counted on video: highly mobile taxa (isopods, polychaetes Macellicephalinae, amphipods, shrimps, fishes), infauna represented only by Lebensspuren, gelatinous zooplankton, small-size organisms (<one cm) and organisms that could not be identified at least to the phylum level. For shrimps and jellyfishes, exceptions were made for near bottom species numerous in some areas.

Because the densities of some species on soft sediment were relatively high it was most optimal to extract individual images (print screen) from the quantitative transects with an interval of 30 s to determine densities. In total 365 digital images were examined using the Photoshop software. A ten-centimetre laser scale was used for calculation of the seafloor surface area on images.

### Data analysis

Statistical analysis was performed in Primer V6 (Clarke & Warwick, 2001).

In order to discern whether depth was driving changes in community composition a cluster analysis was performed based on quantitative transects on the soft substrate. Each quantitative transect was treated as a separate sample. The relative contribution (%) of each species to total abundance on each transect was calculated. Relative abundance data were square-root transformed to reduce the dominance of the most abundant species. The similarity between transects was estimated using the quantitative Bray–Curtis index. Similarity matrixes was used for cluster analyses. Clusters were generated using group averaged linkage. The resulting divisions were tested using SIMPROF permutation tests, for looking statistically significant evidence of genuine clusters in samples which are a priori unstructured. Revealed upper and lower boundaries of depth ranges based on the analysis of quantitative transects were adjusted based on the visual observations of dominance of the habitat-forming taxa. It was done because there were significant sections of the video that didn’t fall on the quantitative transects. Average density per depth range (±standard deviation) of the most abundant soft sediment taxa was calculated in order to quantitatively describe differences between detected zones.

Boundaries of the depth ranges within 1,370–350 m with >90% hard substrate were estimated visually based on video observations of the dominant taxa. At the depths 1,750–1,370 m with 70% hard substrate we separately analysed areas of the soft substrate that were used in the cluster analysis and areas with a hard substrate at which dominant taxa was evaluated only visually based on video observations.

The bathymetric distribution of dominant species was then plotted to visually determine the approximate depths where the largest turnover of dominant species occurred. These are the depths where species dominant over a wide depth range are replaced by other dominants. This information was then used to assess the number of identified species, aggregated into several major taxonomic groups, that occur between determined depths.

## Results

Overall about 170 species of megafauna were recognised on images. 30% of these species were collected using ROV and identified in the laboratory by taxonomic experts (Table S1). Two species of Hexactinellida (genus *Farrea*), three species of Actiniaria (genus *Sicyonis*), one species of Corallimorpharia (Corallimorphidae gen. sp.) and one species of Holothuroidea (*Zygothuria* sp.1) collected using ROV appeared to be new to science and they will be described separately. In addition, three species are assumed to also be new to science based on images and/or video records (one species each of ascidian, antipatharian and benthic siphonophorae), but these cannot be verified without physical samples.

### The vertical distribution of communities

**Figure 2:**
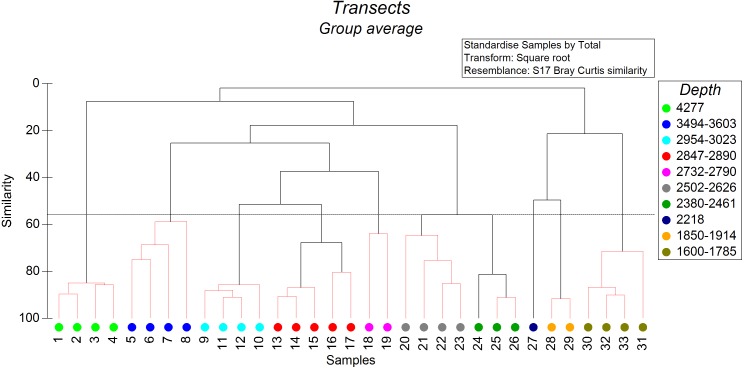
The results of the cluster analysis of soft sediment communities (from quantitative transects) based on the Bray–Curtis similarity index. The colour indicates the depth range of clustered transects. The black dotted horizontal line corresponds to the 56% similarity level—the level at which the distinct groups of megafauna communities were distinguished. Transects that cannot be significantly differentiated based on SIMPROF tests were connected by red dotted lines (*p* < 0.05).

The non-metric cluster analysis revealed ten distinct groups of soft-sediment megabenthic communities distinguished by depth of occurrence (Fig. 2) at the similarity level of 56% (SIMPROF: *R* = 7,438, *p* = 0.001). Transects that cannot be significantly differentiated based on SIMPROF tests were connected on Fig. 2 by red dotted lines. In two cases transects differentiated based on SIMPROF tests were located in a very close depth range, which may indicate possible other factors affecting their community structure.

We distinguished separately the depth zone 1,750–1,370 m with 70% hard substrate where sections of the soft and hard substrate were analysed independently. This zone was characterized by frequent occurrence of the sponge *Farrea* spp on hard substrate (two species of *Farrea* were collected at similar depths by ROV—*F*. *kurilensis* Okada, 1932 and *F*. *aspondyla* Reiswig & Stone, 2013) whereas communities of soft substrate were similar to depth zone 1,830–1,750 m (dominance of the holothurian *Synallactes chuni* Augustin, 1908 and ophiuroids).

Additionally, four depth zones were distinguished within the depth range 1,370–350 m (>90% hard substrate) based on video observations of the dominant taxa.

Characteristics of communities in each depth zone (dominant species on the soft substrate and common species on hard and soft substrate) are given in Table 2 (upper and lower boundaries of depth ranges are given with adjusted based on the visual observations of dominance of the habitat-forming taxa in the whole video footage).

**Table 2:** Characteristics of megafaunal communities at different depths. The depths are arranged up the slope in line with the ROV dives.

**Depth range (m)**	**Dominant species on soft and/or hard substrate**	**Soft substrate (%)**	**Common species on soft substrate**	**Common species on hard substrate**
4,277–4,278	*Kolga kamchatica*	100%	Echiura gen.sp., *Munidopsis* spp., *Zygothuria* sp.1, *Psychropotes* “*raripes* ”, *Astrocles actinodetus*, Enteropneusta gen. sp.1	Demospongiae varia, *Hyalonema* sp*.*, Crinoidea (unstalked)
3,610–3,450	*Scotoplanes kurilensis*	90	*Holascus* sp., Ceriantharia varia, *Umbellula sp.*, *Pennatula* sp*.*, *Munidopsis* spp., *Kolga kamchatica*, *Zygothuria* sp. 1, Cucumariidae gen sp., *Astrocles actinodetus*, Enteropneusta gen. sp.1	Demospongiae varia, *Stylaster* spp., Crinoidea (stalked and unstalked), Ascidia varia
3,030–2,910	*Ophiura bathybia, Scotoplanes kurilensis*	90	Ceriantharia varia, *Paelopatides solea*	Demospongiae varia, *Stylaster* spp., Brachiopoda varia, Crinoidea (stalked and unstalked), Ascidia varia
2,910–2,790	*Scotoplanes kurilensis, Ophiura bathybia*, Crangonidae gen. sp.	80	Ceriantharia varia, *Paelopatides solea*	Demospongiae varia, Rossellidae varia, *Stylaster* spp., Alcyonacea varia, *Ophiocantha* spp., Crinoidea (stalked and unstalked), Ascidia varia
2,790–2,650	Crangonidae gen. sp.	80	*Paelopatides solea*, *Ophiura bathybia*	Demospongiae varia, *Ophiocantha* spp., Crinoidea (stalked and unstalked), Ascidia varia
2,650–2,470	*Paelopatides solea*, Crangonidae gen. sp.	80	Rhopalonematidae gen. sp.	Demospongiae varia, Alcyonacea varia, *Astrochele laevis*, Crinoidea (stalked and unstalked), Ascidia varia
2,470–2,290	*Paelopatides solea*, Crangonidae gen. sp., Rhopalonematidae gen. sp.	70	Torquaratoridae gen. sp., *Pannychia* aff. *moseleyi*	Demospongiae varia, Alcyonacea varia, Brachiopoda varia, *Ophiura* spp., *Ophiacantha* spp., Crinoidea (unstalked), Ascidia varia
2,290–2,130	Rhopalonematidae gen. sp., Torquaratoridae gen. sp.	60	*Paelopatides solea*, *Pannychia* aff. *moseleyi*, Crangonidae gen. sp.	Demospongiae varia, Rossellidae varia, Alcyonacea varia, Brachiopoda varia, *Ophiura* spp., *Ophiacantha* spp., Crinoidea (unstalked), Torquaratoridae gen. sp., Ascidia varia
2,130–1,830	Torquaratoridae gen. sp.	50	*Pannychia* aff. *moseleyi*, *Synallactes chuni*, *Ophiura* spp., *Ophiocantha* spp., Rhopalonematidae gen. sp.	Demospongiae varia, Hexactinellida varia, Alcyonacea varia, Brachiopoda varia, *Synallactes chuni*, *Ophiura* spp., *Ophiacantha* spp., Crinoidea (unstalked), Torquaratoridae gen. sp., Ascidia varia
1,830–1,750	*Synallactes chuni*, *Ophiura* spp., *Ophiocantha* spp.	40	Torquaratoridae gen. sp., Ceriantharia varia	Demospongiae varia, *Farrea* spp., *Stylaster* spp., Brachiopoda varia, *Synallactes chuni*, *Ophiura* spp., *Ophiacantha* spp., Crinoidea (unstalked), Ascidia varia
1,750–1,370	*Ophiura* spp., *Ophiocantha* spp., *Synallactes chuni*, *Farrea* spp.	30 (stones and dead *Farrea*)	Torquaratoridae gen. sp., *Pannychia* aff. *moseleyi*	Demospongiae varia, Rossellidae varia, *Stylaster* spp., *Paragorgia* sp. 1, *Munidopsis* spp., Galatheidae gen. spp., Lithodidae gen. spp., Brachiopoda varia, *Ophiophthalmus* sp., *Ophiura* spp., *Ophiacantha* spp., *Pteraster* sp. 2, Goniasteridae gen.sp.2, *Hydrasterias* sp., Crinoidea (unstalked), Ascidia varia
1,370–720	*Farrea* spp*.*, *Ophiura* spp., *Ophiocantha* spp.	10 (stones and dead Farrea)	*Ophiura* spp., *Ophiocantha* spp.	Demospongiae varia, Rossellidae varia, *Heterochone* sp., *Heteropolypus ritteri*, *Paragorgia* sp. 1, *Munidopsis* spp., Galatheidae gen. spp., Lithodidae gen. spp., *Psolus* spp., *Ophiura* spp., *Ophiacantha* spp., *Ophiophthalmus* sp., Goniasteridae gen. sp. 2 and sp.3, Crinoidea (unstalked), Ascidia varia
720–440	*Heteropolypus ritteri*	0	–	Demospongiae varia, *Farrea* spp., *Pinulasma fistulosum*, Rossellidae varia *, Tritonia* cf. *diomedea,* Galatheidae gen. spp., Lithodidae gen. spp., *Psolus* spp., Goniasteridae gen. sp.2
440–390	*Heteropolypus ritteri, Corallimorphus pilatus*	0	–	Demospongiae varia, *Caryophylliidae gen. sp. 1*, Corallimorphidae gen. sp., Polyplacophora varia, *Tritonia* cf. *diomedea*, *Terebratulina* cf. *kiiensis*, Goniasteridae gen. sp.2., *Solaster* sp.
390–350	*Epizoanthus* sp.	0	–	Demospongiae varia, *Actinothoe* sp.,*Caryophylliidae gen. sp. 1*, Corallimorphidae gen. sp., *Heteropolypus ritteri*, Polyplacophora varia, *Terebratulina* cf. *kiiensis*, *Ophiopholis* cf*. japonica*, Goniasteridae gen. sp. 2 and sp.3, *Pteraster* sp. 2, *Crossaster papposus*

**Figure 3:**
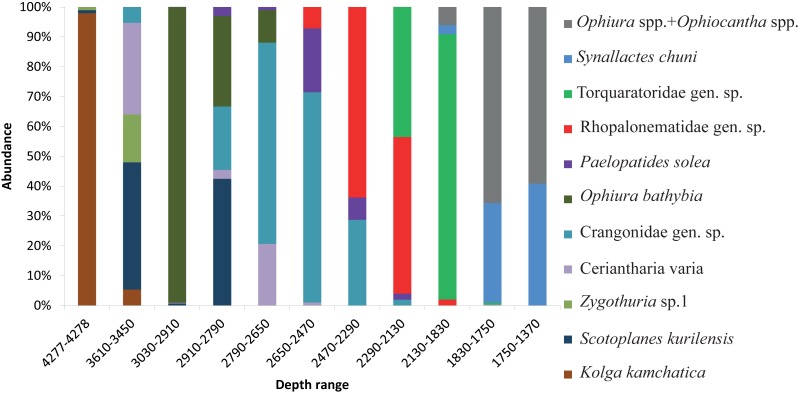
The contribution to total abundance (in %) of the most abundant taxa in each of the clustered depth zones.

**Figure 4:**
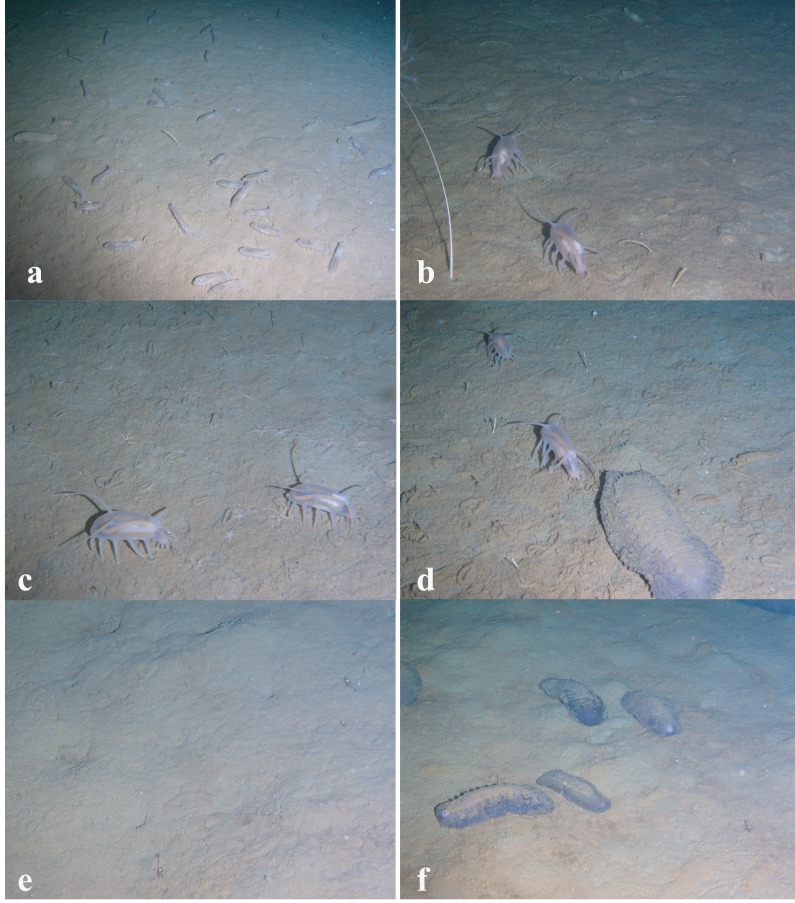
Images of dominant megafauna at different depths. (A) 4,277–4,278 m, the holothurian *Kolga kamchatica.* (B) 3,610–3,450 m, the holothurian *Scotoplanes kurilensis.* (C) 3,030–2,910 m, the ophiuroid *Ophiura bathybia* and the holothurian *S. kurilensis.* (D) 2,910–2,790 m, the holothurian *S. kurilensis,* the** ophiuroid *O. bathybia* and the shrimp Crangonidae gen. sp. (E) 2,790–2,650 m, the shrimp Crangonidae gen.sp. (F) 2,650–2,470 m, the holothurian *Paelopatides solea* and the shrimp Crangonidae gen. sp.

**Figure 5:**
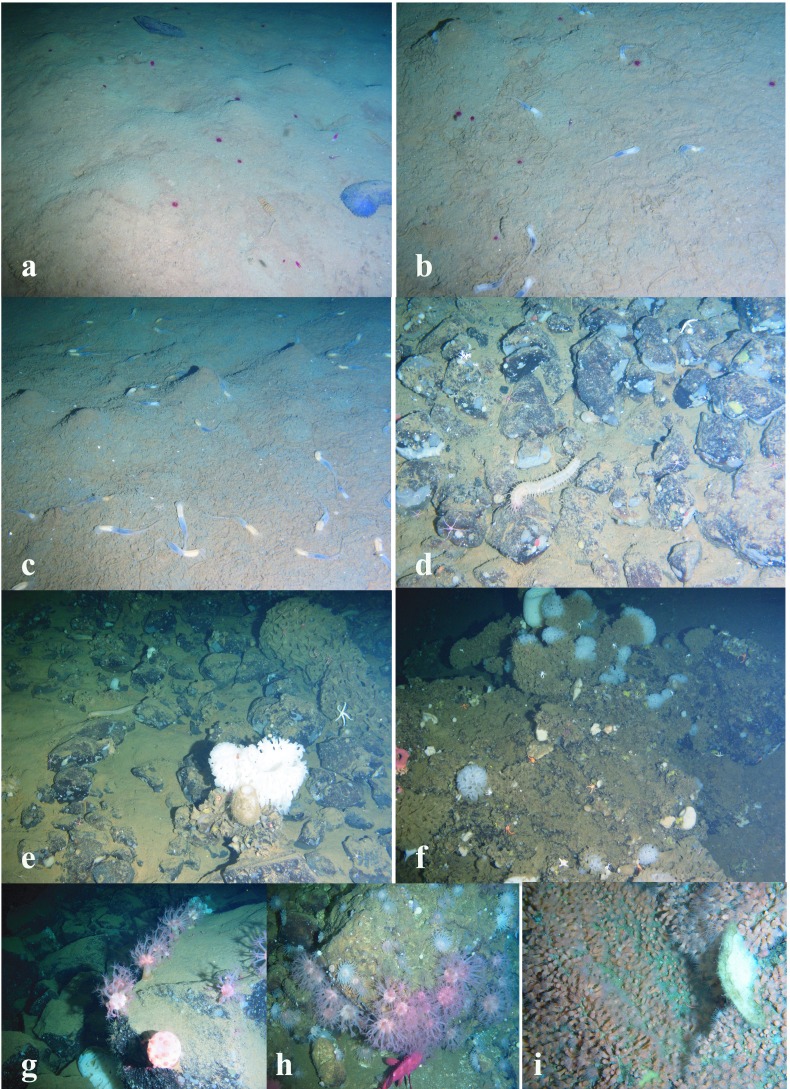
Images of dominant megafauna at different depths (continued). (A) 2,470–2,290 m, the holothurian *Paelopatides solea*, the shrimp Crangonidae gen. sp. and the jellyfish Rhopalonematidae gen. sp. (B) 2,290–2,130 m, the jellyfish Rhopalonematidae gen. sp. and the enteropneust Torquaratoridae gen. sp. (C) 2,130–1,830 m, the enteropneust Torquaratoridae gen. sp. (D) 1,830–1,750 m, the holothurian *Synallactes chuni* and the ophiuroid (*Ophiura* spp. and *Ophiocantha* spp.). (E) 1,750–1,370 m, ophiuroids, the holothurian *S. chuni* and the glass sponge *Farrea* spp. (F) 1,370–720 m, the sponge *Farrea* spp.** and ophiuroids. (G) 720–440 m, the cnidarian *Heteropolypus ritteri.* (H) 440–390 m, the cnidarian *H. ritteri* and *Corallimorphus pilatus.* (I) 390–350 m, the cnidarian *Epizoanthus sp.*

The dominant taxa changed with depth in the following order: the holothurian *Kolga kamchatica* Rogacheva, 2012 at the maximum depth (4,277–4,278 m); the holothurian *Scotoplanes kurilensis* Gebruk, 1983 was dominant or abundant at depths 3,610–2,790 m; the ophiuroid *Ophiura bathybia* Clark, 1911 sharply dominated at depths 3,030–2,910 m; benthic crangonid shrimps dominated at 2,910–2,290 m with a peak at 2,790–2,470 m; the holothurian *Paelopatides solea* Baranova, 1955 at 2,650–2,290 m; benthic jellyfish at 2,470–2,130 m; enteropneusts at 2,290–1,830 m; the holothurian *Synallactes chuni* and ophiuroid of the genera *Ophiura* and *Ophiocantha* at 1,830–1,370 m; the glass sponge *Farrea* spp. at 1,750–1,370 m. At the depths 1,370–720 m almost all benthic epifauna was associated with live or dead colonies of *Farrea* spp.*,* except of areas of the soft substrate (10% of the total area) dominated by the ophiuroids *Ophiura* and *Ophiocantha*. Depths 720–390 m were dominated by the coral *Heteropolypus ritteri* Nutting, 1909. The corallimorpharian *Corallimorphus pilatus* Fautin, White & Pearson, 2002 was the dominant at 440–390 m. At the minimum in our study depth 390–350 m, the zoantharian *Epizoanthus* sp. was the dominant taxon. Contribution to the abundance (in %) per depth zone of the most abundant taxa, based on analysis of quantitative transects, is presented in Fig. 3 and **Table 3. Densities of the most abundant taxa are given in **Table 3**. Characteristic images of all distinguished depth zones with dominant species are presented in Figs. 4 and 5.

**Table 3:** The mean densities (ind m^−2^) ± standard deviations and the contribution to total abundance (in %) of the most abundant taxa in different depth zones. The dominant taxa are shaded grey.

**Depth range**	4,277–4,278	3,610–3,450	3,030–2,910	2,910–2,790	2,790–2,650	2,650–2,470	2,470–2,290	2,290–2,130	2,130–1,830	1,830–1,750	1,750–1,370
*Kolga kamchatica*	94%	4%	0	0	0	0	0	0	0	0	0
	16.7 ± 8.2	0.1 ± 0.2	0	0	0	0	0	0	0	0	0
*Scotoplanes kurilensis*	1%	32%	0.7%	42%	0	0	0	0	0	0	0
	0.0 ± 0.0	1.1 ± 0.9	0.8 ± 1.0	3.5 ± 3.3	0	0	0	0	0	0	0
*Zygothuria* sp.	1%	12%	0	0	0	0	0	0	0	0	0
	0.0 ± 0.0	0.3 ± 0.5	0	0	0	0	0	0	0	0	0
Ceriantharia varia	0	23%	0.2%	3%	19%	1%	0	0	0	0.3%	0
	0	0.1 ± 0.3	0.2 ± 0.5	0.0 ± 0.0	0.1 ± 0.3	0.0 ± 0,0	0	0	0	0.2 ± 0.0	0
Crangonidae gen. sp.	0	4%	0.05%	21%	62%	69%	27%	2%	0	0	0
	0	0.0 ± 0.0	0.0 ± 0.0	0.2 ± 0.7	0.4 ± 0.5	1.1 ± 0.9	1.1 ± 1.5	0.6 ± 1.1	0	0	0
*Ophiura bathybia*	0	0	99%	30%	10%	0	0	0	0	0	0
	0	0	74.2 ± 24.0	3.7 ± 3.1	0.2 ± 0.5	0	0	0	0	0	0
*Paelopatides solea*	0	0	0.05%	3%	1%	21%	7%	2%	0	0	0
	0	0	0.0 ± 0.0	0.1 ± 0.3	0.0 ± 0.0	0.5 ± 0.8	0.6 ± 0.7	0.2 ± 0.4	0	0	0
Rhopalonematidae gen. sp.	0	0	0	0	0	7%	60%	53%	2%	0	0
	0	0	0	0	0	0.4 ± 0.7	3.3 ± 2.7	4.2 ± 5.5	0,1 ± 0,3	0	0
Torquaratoridae gen. sp.	0	0	0	0	0	0	0	44%	88%	0.6%	0
	0	0	0	0	0	0	0	8.8 ± 4.1	11.9 ± 4.3	0.6 ± 0,9	0
Synallactes chuni	0	0	0	0	0	0	0	0	3%	33%	40%
	0	0	0	0	0	0	0	0	0.5 ± 0.5	1.4 ± 1.5	0.4 ± 0.5
Ophiura spp.+Ophiocantha spp.	0	0	0	0	0	0	0	0.05%	6%	65%	58%
	0	0	0	0	0	0	0	1.1 ± 1.0	1.1 ± 1.3	3.8 ± 5.9	5.2 ± 3.2

### The depths with the largest community changes

**Figure 6:**
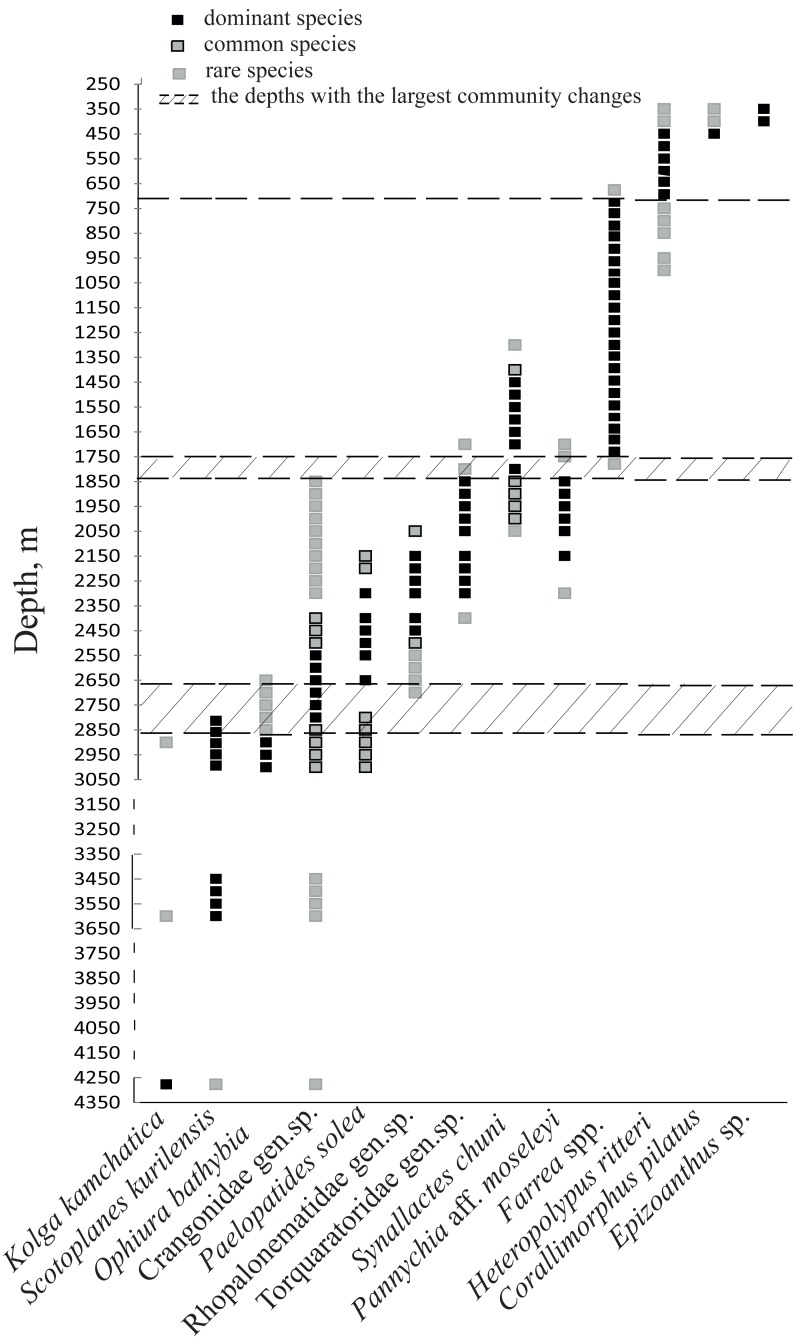
The bathymetric distribution of the dominant megafaunal species. Each symbol corresponds to the occurrence of a species at a certain depth based on video. The three types of markers show the species status according to the following scale: “dominant”—was more abundant than any other species, “common”—occurred often and was relatively abundant and “rare”—was only met a few times). The dotted lines show the depths with the largest megafauna community changes (the depths of the largest turnover of dominant taxa).

Based on the vertical distribution of dominant megafaunal taxa, the depths of the largest community changes, or the largest turnover of dominant species, were revealed (Fig. 6). The first pronounced change occurred at ∼2,790 m depth and was identified by the replacement of the holothurian *Scotoplanes kurilensis* by other species of holothurians, enteropneusts, benthic jellyfish and shrimps. The second pronounced change occurred at ∼1,750 m: here the glass sponge *Farrea* spp. took the role of the landscape determining species. The third change at ∼720 m was associated with the replacement of glass sponges by soft corals. We also suggest that a community change may have occurred at depths between 3,650–4,200 m (but a lack of observations between 4,277–3,650 m limited more precise zoning). This change was indicated by the replacement of the holothurian *Kolga kamchatica* by the holothurian *Scotoplanes kurilensis.*

**Figure 7:**
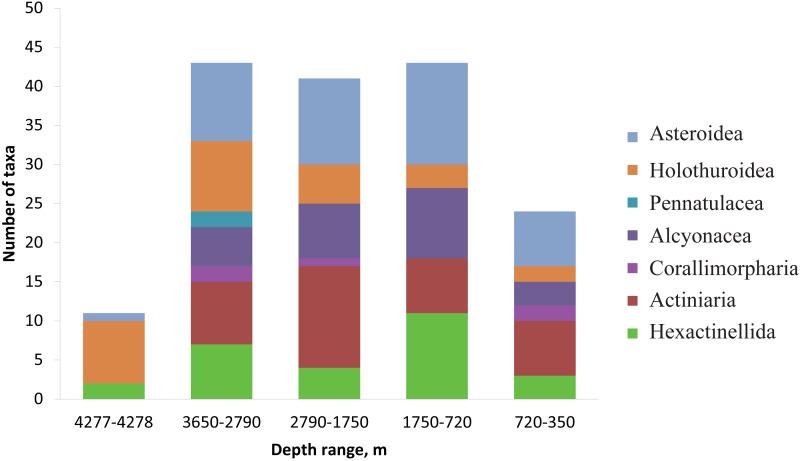
The number of identified species, aggregated into several major taxonomic groups, that occur between depths with the largest community changes.

The number of identified species, aggregated into several major taxonomic groups, that occur between depths with the largest community changes is presented in Fig. 7. The highest number of species was found at depths 3,650–2,790 m, the lowest—at 4,277–4,278 m. The taxon with the highest species richness at the maximum depth 4,277–4,278 m was Holothuroidea (eight species). The relatively high number of holothurian species was also at 3,650–2,790 m (nine species). The Order Pennatulacea was recorded only at 3,650–2,790 m (two species). The highest diversity of actiniarians was at depths 2,790–1,750 m (14 species). Alcyonacean corals showed the highest species richness at 1,750–720 m (not less than 9 species). The highest species richness of hexactinellid sponges was at 1,750–720 m (not less than ten species).

## Discussion

In the present study, the vertical distribution of benthic megafaunal communities was described quantitatively for the first time on the slope of the Volcanologists Massif, in the south-west Bering Sea and adjacent Komandorsky Basin in the depth range 4,278–349 m. We were able to identify approximately 170 species, based on the photo and video surveys together with samples taken by ROV. Fifteen depth zones were revealed. Seven species collected using ROV appeared new to science and will be described separately. In addition, three species are assumed to be new to science based on images and/or video records, but further research and sampling using ROV in this area are required for taxonomic clarification.

### Soft sediment communities

The greatest depths of the Komandorsky Basin (4,278 m) were dominated by holothurians (eight species) with the prevailing of the elpidiid *Kolga kamchatica*. Aggregations of small elpidiid holothurians *Kolga* in the abyssal zone is a phenomenon observed in several areas of the ocean. For example, *Kolga nana* Théel, 1879 aggregations were found in the Charlie-Gibbs Fracture Zone (Gebruk & Krylova, 2013) with densities of up to 76 ind/m^2^ and in the Porcupine Seabight in the North Atlantic (Billett & Hansen, 1982) with a mean density of 34 ind/m^2^ at depths at around 4,000 m. Specimens of *K. nana* were observed in the Charlie-Gibbs Fracture Zone feeding on the soft sediment mainly in flat and gentle slope areas (Rogacheva, 2012). Another species of this genus*, K. hyalina* Danielssen & Koren, 1879*,* was abundant in the Central Arctic Basins with densities significantly dependent on seasonal ice-algae supply to the seafloor (Rybakova et al., 2019). The density of *K. kamchatica* in our study (17 ind/m^2^) was lower than in the mentioned reports, although it was still relatively high. Another elpidiid holothurian, *Scotoplanes kurilensis*, with lower mean density 1–4 ind/m^2^, was abundant at depths 3,610–2,790 m. In general, elpidiid holothurians can reach high abundances, especially in seafloor depressions in the abyssal zone. Rowe (1971) regarded aggregations of elpidiid holothurians as a typical feature of canyons which trap organic matter that attracts deposit-feeders. However, aggregations of elpidiid holothurians are not exclusive to seafloor depressions: small elpidiids commonly aggregate in areas of accumulation of organic matter on the seafloor (Gebruk, 1990; Billett, 1991; Roberts et al., 2000; Gebruk et al., 2013). Amaro et al. (2010) showed on the example of infaunal molpadiid holothurian *Molpadia musculus* Risso, 1826 from the Nazare Canyon (NE Atlantic) the key functional role of holothurians in the degradation of organic matter from the sediments and its redistribution and availability for other fauna.

Generally, depths greater 3,500 m in our study were characterized by a high diversity of holothurians (11 species), with one species usually dominant at any given depth. Deposit-feeding holothurians are known to have different feeding strategies and activity (Roberts et al., 2000). Feeding-guilds and niche separation optimize the use of food resources. Studies in various regions showed that holothurians, especially elpidiids, are able to react rapidly to the changes in food supply (Billett et al., 2001; Billet et al., 2010; Gutt et al., 2011; Kuhnz et al., 2014; Huffard et al., 2016).

At 3,610–3,450 m depth, a high diversity of suspension feeders associated with soft substrate was notable, including the hexactinellid sponge *Holascus* sp., some unidentified species of ceriantharians and two species of Pennatulacea. It can be suggested that this is related to peculiarities of near-bottom hydrodynamics in this area. The growth of the relative importance of sessile suspension feeders (sponges and ceriantharians) deeper than 3,000 m was mentioned by Kuznetzov (1964) based on limited data from the Central and Komandorsky Basins of the Bering Sea: these species developed specific adaptations to the soft-clay habitats, such as raising their bodies above the sediment and the ability to utilize food from the near bottom water layers.

The ophiuroid *Ophiura bathybia* formed aggregations at 3,030–2,910 m depth with a very high mean density (74 ind/m^2^). Many ophiuroid taxa are predators (Warner, 1982). Perhaps local sedimentation or hydrological conditions may affect food concentration for them in this area.

At 2,790–2,650 m depth the species diversity and abundance of megafauna were low. Unidentified shrimps of the family Crangonidae were the most notable and abundant soft-sediment megafauna. The hard substrate together with the rare synallactid holothurians and corals at this depth were covered with a considerable sediment layer. We hypothesize that this was an effect of active re-deposition or rapid sedimentation causing decrease in abundance and diversity of megafauna.

On the lower slope, at depths from 3,000 m to 2,150 m, the synallactid holothurian, *Paelopatides solea*, played a significant role. Numerically they were not dominant, however owing to big size, their contribution to the biomass must have been significant. It means that they may play an important role in ecosystem functioning at that depth range utilizing organic matter from the sediment.

Communities with numerical dominance of benthopelagic rhopalonematid jellyfish at depths >1,500 m (2,470–2,130 m in the present study) to our knowledge have not been reported before. Traditional methods of benthos investigations (trawls and grabs) do not allow the detection of aggregations of gelatinous organisms at the seafloor, because such organisms are usually destroyed in catches. Visual seafloor inspection can resolve this problem. Different observations at depths of up to one and a half thousand meters suggest that deep-sea medusae can be abundant near the bottom (Larson et al., 1992; Matsumoto, Baxter & Chen, 1997; Grange et al., 2017). It can be suggested that aggregations of jellyfish at the seafloor are related to the local near-bottom current regime, apparently increasing food availability through resuspension of organic matter (Larson et al., 1992). Soft sediment megafauna communities dominated by torquaratorid enteropneusts (at the depths 2,130–1,830 m) have not been reported before in the deep-sea either. Deep-sea enteropneusts are also usually gelatinous and easily destroyed in trawl and grab catches. With increased use of ROVs, enteropneusts have been recorded in different deep ocean basins (Priede et al., 2012). Deep-sea enteropneusts are still poorly known, however their function in benthic ecosystems can be important, especially in organic matter processing and surficial bioturbation (Jones et al., 2013). Deep-water enteropneusts feed on the sediment surface, collecting detritus from the substrate, apparently with little or no selectivity as they crawl forward leaving behind a faecal trail of undigestible presumably mineralised material (Holland et al., 2009). Some species have demonstrated the ability to use near-bottom currents to move between feeding grounds in a controlled manner through changes in body posture (Osborn et al., 2012; Jones et al., 2013). Estimation of the area of their traces in the northern part of the Mid-Atlantic Ridge at around 2,500 m depth revealed that enteropneusts contribute significantly to the surficial deposit feeding (Jones et al., 2013). This was unexpected because local deposit feeding megafaunal density was dominated by holothurians, with only a relatively small proportion of enteropneusts (Jones et al., 2013). According to our observations, the mean density of enteropneusts was unusually high reaching 12 ind m^−2^ at depths 2,130–1,830 m (Priede et al., 2012). The highest density ever previously recorded (for a mixture of Australian torquaratorid species) was 29 worms per 1,000 m^2^ (Anderson, Przeslawski & Tran, 2011). The highest density found during a 15-year study of *Tergivelum baldwinae* Holland et al., 2009 from the Pacific Ocean was two worms per 1,000 m^2^ (Smith, Holland & Ruhl, 2005). In our study area, this taxon definitely must play a major role in the utilization of organic matter at the seafloor.

Small areas of soft sediment (10% of the total area) at depths 1,830–1,370 m were inhabited mainly by the holothurians *Synallactes chuni* and *Pannychia* aff. *moseleyi* Théel, 1882 and some species of ophiuroids. Notably, these species of holothurians and ophiuroids occurred also on hard substrate in that area which may indicate that they are ubiquitous, with no specific adaptations to soft sediment.

### Hard substrate communities

The bottom of the Komandorsky Basin was 100% sediment covered, with rare occurrences of colonies of the hexactinellid sponge, *Farrea* sp. 1, with associated fauna. From the base of the slope up to 1,830 m depth, the area covered by sediment smoothly decreased to 50%, while the area of hard substrate increased. Accordingly, the diversity of species living on rocky outcrops increased, including sessile forms of corals, actiniarians, sponges, ascidians and others. Hard substrate provides more heterogeneous habitat as compared to soft sediment, additionally some organisms use sessile megafaunal species (e.g., sponges, corals) as substrates for attachment (e.g., ophiuroids living on gorgonian corals) (Levin et al., 2001; BuhlMortensen et al., 2010). Some corals and sponges modify habitats through their physical presence. They provide living space, alter physical conditions and affect biological interactions, considerably enhancing diversity (Felley, Vecchione & Wilson, 2008; Miller et al., 2012; Gebruk & Krylova, 2013).

At 1,830 to 1,370 m depth, the area of the hard substrate increased up to 90%. Rocky outcrops alternated with small areas of soft sediment. The deposit feeding holothurians, *Synallactes chuni* and *Pannychia* aff. *moseleyi,* and some species of ophiuroids occurred together with highly diverse sessile suspension feeding fauna on rocks covered by a thin layer of sediment. The ratio of sessile suspension feeders is known to increase on rocks owing to increased active water circulation (Kuznetzov, 1964; Witman & Dayton, 2001; Tyler, 2003; Miller & Etter, 2008; Miller & Etter, 2011). Starting from a depth of 1,750 m, the hard substrates were covered by live and dead colonies of the hexactinellid sponge, *Farrea* spp*.,* with associated fauna. At 720 m this community was sharply replaced by an assemblage of cnidarians, including *Heteropolypus ritteri*, *Corallimorphus pilatus*, *Epizoanthus* sp. and various actiniarians. From a depth of about 1,750 m, the slope became steeper, possibly affecting water circulation and providing a habitat favourable for suspension feeders. The exact reasons of replacement of sponges by cnidarians remain unknown.

It is important to note that, based on the video or photo images, species could not be equally identified across all taxa, thus limiting the comparison of species diversity at different depths.

### Generalized patterns of the vertical zonation

Depths with the largest community changes identified in our study as depths of the largest turnover of dominant taxa, in general correspond to the patterns of vertical zonation recognized by other authors (Carney, Haedrich & Rowe, 1983; Mironov, 1986; Howell, Billett & Tyler, 2002). Major faunal changes occurred at the transition between bathyal and abyssal zones, at ∼2,790 m depth. At this depth, the abyssal community dominated by the deposit-feeding holothurian *Scotoplanes* was replaced by the bathyal/slope communities of different feeding types (with several dominant species characterized by different feeding types) that may reflect changes in the quality and quantity of food resources at the bottom. The boundary between the abyssal and bathyal zones at a depth of ∼3,000 m was commonly noticed in previous works, however some authors recognized this boundary closer to 2,000 m (Gage & Tyler, 1991) or 4,000 m (Thurman, 1985). In the scheme of the vertical zonation suggested by Belyaev et al. (1959), the bathyal is defined as a depth zone between 200 m and 3,000 m, with a zone between 2,500 m and 3,500 m considered as the transition between the bathyal and abyssal zones. The boundary between the abyssal and bathyal zones can vary around the world based among other factors on geomorphological features of the seabed (Menzies, George & Rowe, 1973). Also, the depth at which this boundary stands out significantly depends on the methodology used. Within the abyssal zone we also identified considerable community change between the *Scotoplanes* dominated and *Kolga* dominated communities at depths between 3,650 m and 4,277 m.

The zoning of the bathyal zone also differs in published literature. Many authors distinguish the lower, middle and upper bathyal/slope based on the distribution of species or communities, however the depth boundaries of these horizons differ (Howell, Billett & Tyler, 2002). In the present study we distinguished a variety of communities dominated by soft or hard substrate fauna and deposit-feeding or suspension-feeding species at depths from ∼2,790 m to ∼1,750 m which may correspond to a lower bathyal zone. The mid bathyal zone we recognized between ∼1,750 m and ∼720 m, dominated by suspension-feeding hexactinellid sponges associated with the increased availability of hard substrate. The upper bathyal zone appeared to occur between ∼720 m and 350 m, dominated by suspension-feeding cnidarians on hard substrate. In distinguishing the sub-zones within the bathyal zone, we followed the approach of Belyaev et al. (1959).

The relatively high species richness in our study occurred between the depths of 3,650–720 compared to depth ranges of 4,277–4,278 m and 720–350 m. The peak in species richness occurs at different depths in different World Ocean regions (Gebruk, Budaeva & King, 2010). Our results are concordant with several other authors who observed a peak at around 1,500–3,000 m (Rex, 1981; Howell, Billett & Tyler, 2002; Rex & Etter, 2010).

Thus, the vertical distribution of megafauna communities on the northern slope of the Volcanologists Massif (in the south-western Bering Sea) based on the video survey was examined for the first time. We revealed a distinct pattern of vertical zonation. Fifteen types of megafaunal communities, corelated with particular depth ranges, were distinguished. The largest community changes occurred at depths of ∼2,790 m, ∼1,750 m and ∼720 m. These depths correspond to transitions from the abyssal to the bathyal zone, and to transitions between the lower, middle and upper bathyal zones, as shown in a range of publications by different authors. Our results contribute towards better understanding of the regional patterns of vertical faunal zonation in the World Ocean.

##  Supplemental Information

10.7717/peerj.8628/supp-1 Data S1 Images of megafauna identified during 75th cruise of the RV *Akademik M.A. Lavrentyev* on video

10.7717/peerj.8628/supp-2 Table S1 Complete list of taxa identified on video and in collected material during 75th cruise of the RV *Akademik M.A. Lavrentyev* Taxa identified based on collected material are marked by *.
Abundance of all identified species on hard and soft substrate was evaluated visually based on a four-point: 4 - dominant species, 3 - abundant species, 2 - common species, 1 - rare species.

10.7717/peerj.8628/supp-3 Table S2 Soft sediment megafaunal species abundance on quantitative transects selected from five dives of ROV *Comanch 18* during 75th cruise of the RV *Akademik M.A. Lavrentyev*

## Additional Information and Declarations

### Data Availability

The following information was supplied regarding data availability: The raw data are available in the Supplemental Files.
