# Supplementary material for: Vertical distribution of megafauna on the Bering Sea slope based on ROV survey
Source: PeerJ. 2020 Mar 2;8:e8628. doi: 10.7717/peerj.8628 (PMC7058103; doi:10.7717/peerj.8628)

Data S1:

Images of megafauna identified during 75th cruise of the RV *Akademik M.A. Lavrentyev* on video.

Porifera

Demospongiae

Cladorhiza corona

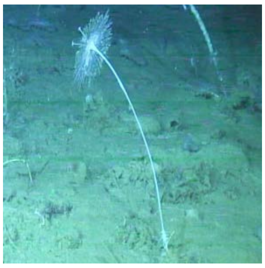

<u>Hexactinellida</u>

Hyalonema sp.

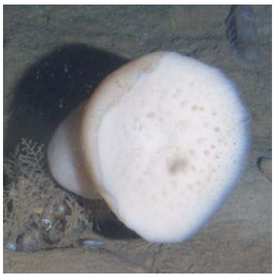

Holascus sp.

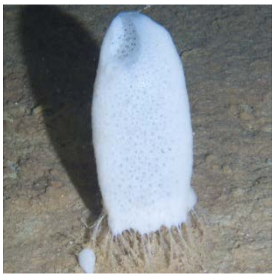

Trachycaulus sp. (stalk)

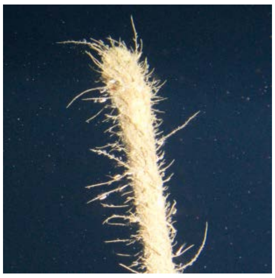

Caulophacus sp.

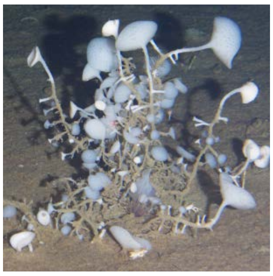

Acanthascus (*Acanthascus*) sp.

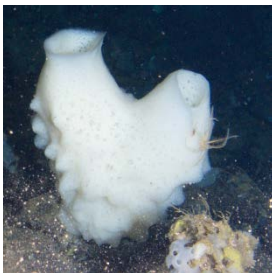

Acanthascus (*Rhabdocalyptus*) sp.

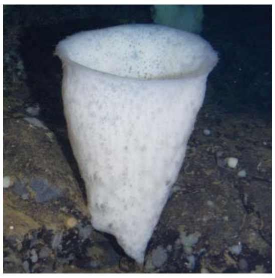

Acanthascus (*Staurocalyptus*) sp.

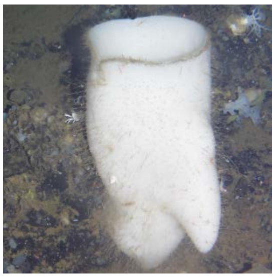

Farrea sp. 1

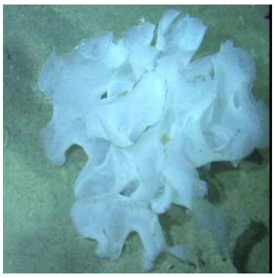

Farrea spp.

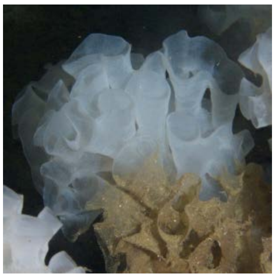

Pinulasma fistulosum

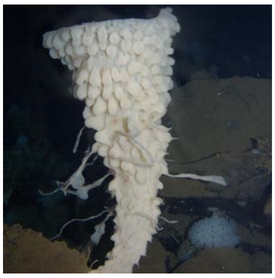

cf. Heterochone

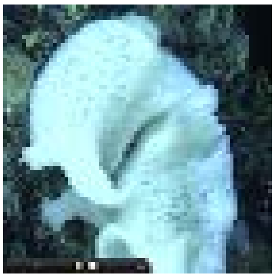

Ctenophora

Ctenophora gen. sp. (benthic form)

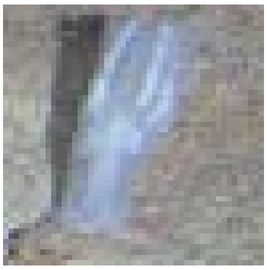

Cnidaria

Hydrozoa

Stylaster spp. *Candelabrum* sp. *Branchiocerianthus imperator* Rhodaliidae gen. sp.

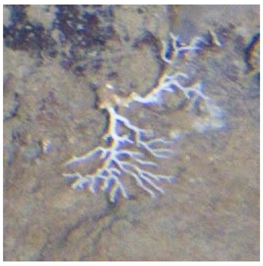
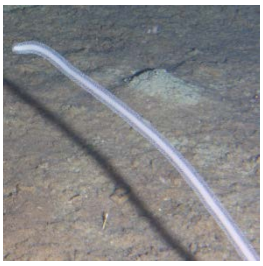
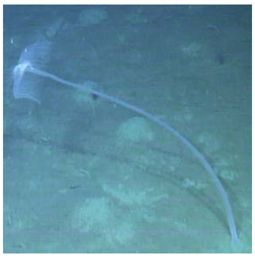
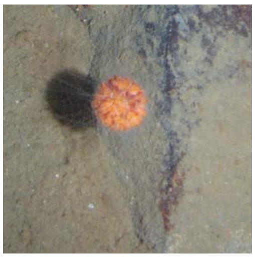

Rhopalonematidae gen. sp.

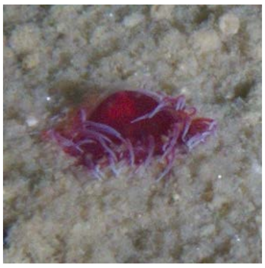

Ceriantharia

Botrucnidiferidae gen. sp.

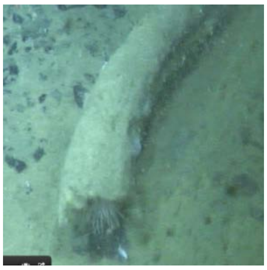

Octocorallia

Alcyonacea

Radicipes sp.

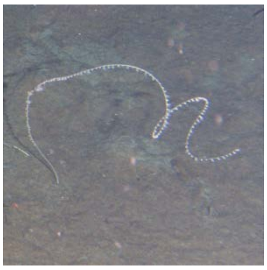

Chrysogorgia sp.

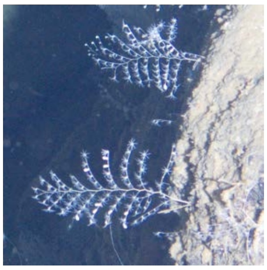

Isidella sp.

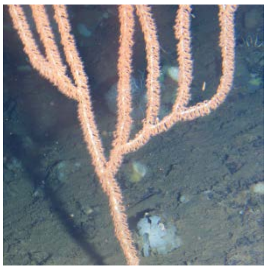

Thouarella sp.

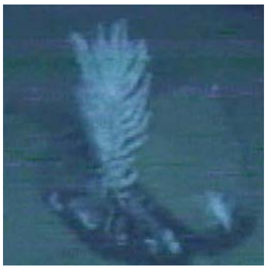

Plumarella spicata

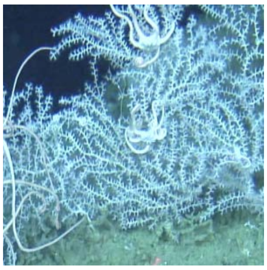

Paragorgia sp.1

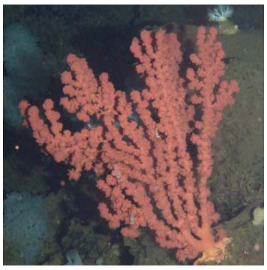

Paragorgia sp.2

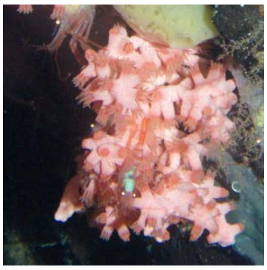

Gersemia sp.

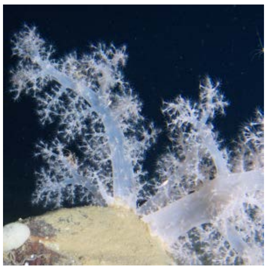

Heteropolypus ritteri

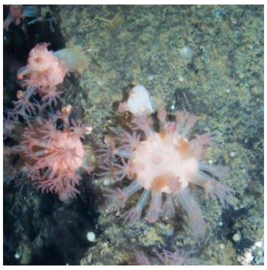

Anthomastus sp.

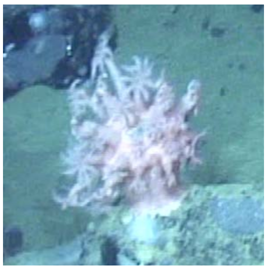

Pseudoanthomastus sp.

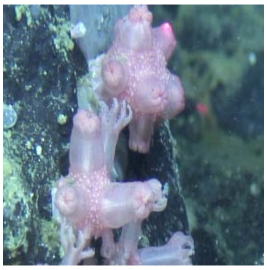

Stolonifera gen. sp.

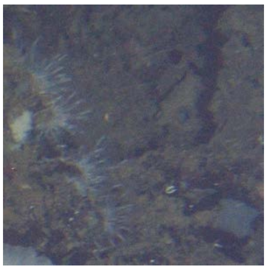

Pennatulacea

Umbellula sp.

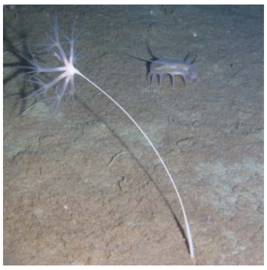

Pennatula sp.

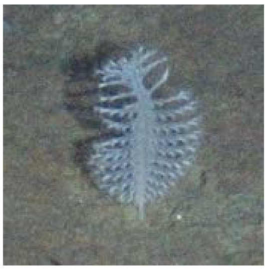

Hexacorallia

Actiniaria

Actinernus robustus

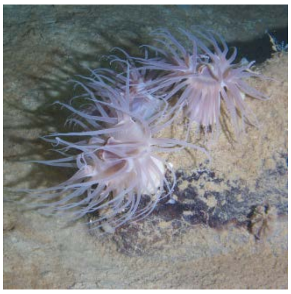

Liponema brevicorne

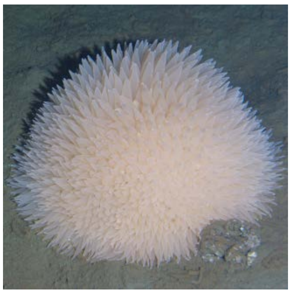

cf. *Halcampoides* sp.

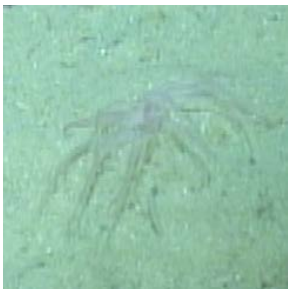

Sicyonis sp.1

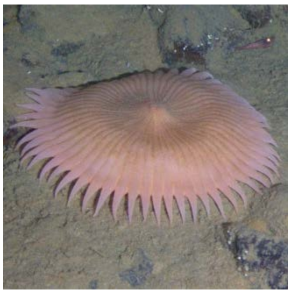

Sicyonis sp.2

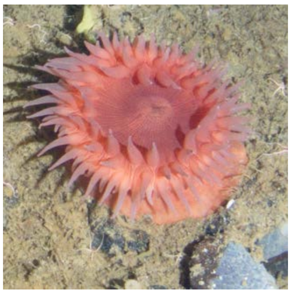

Sicyonis sp.3

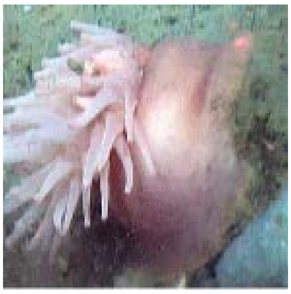

cf. *Actinostola faeculenta*

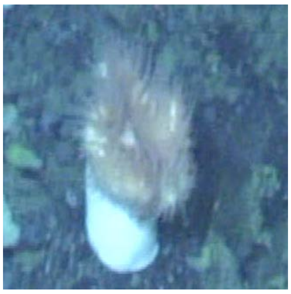

Actinostola sp.1

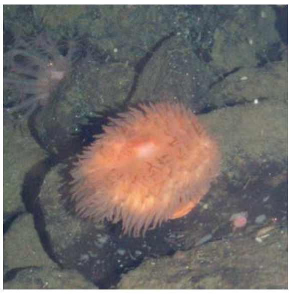

cf. *Actinostola* sp.2

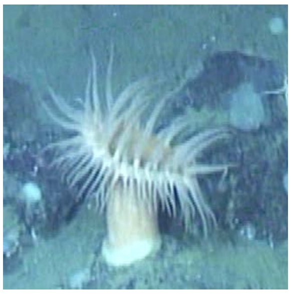

Stomphia sp.

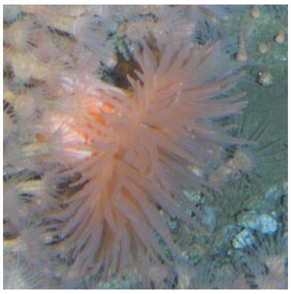

Amphianthus cf. *bathybium*

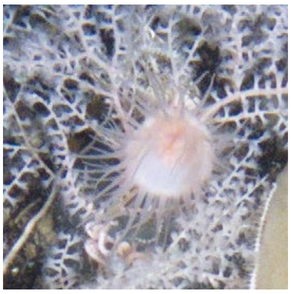

cf. *Amphianthus* sp.

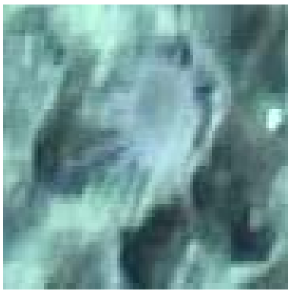

Edwardsiella sp.

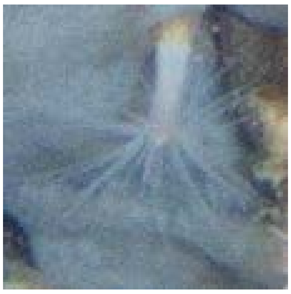

Edwardsiidae gen. sp.

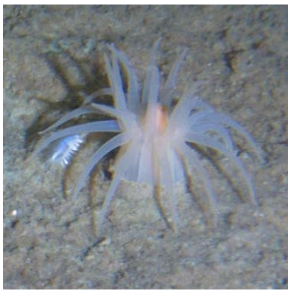

Actinothoe sp.

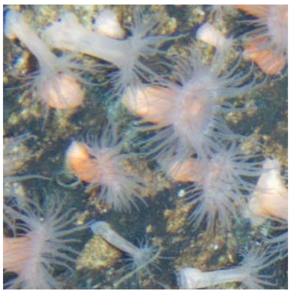

cf. *Hormathia* sp.

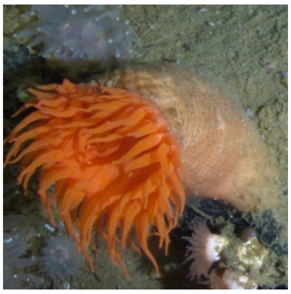

Hormathiidae gen. sp. 1

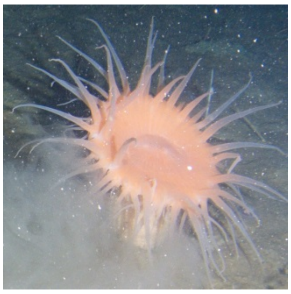

Hormathiidae gen. sp. 2

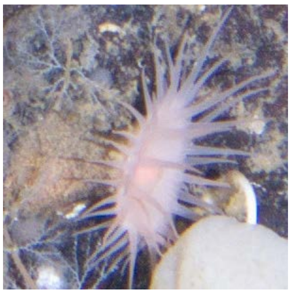

cf. Exocoelactinidae gen. sp.

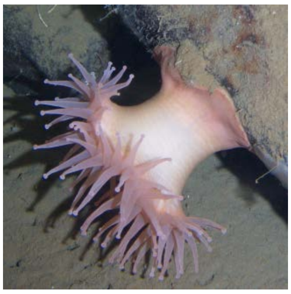

Actiniaria gen. sp. 1

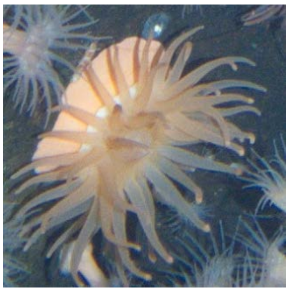

Actiniaria gen. sp. 2

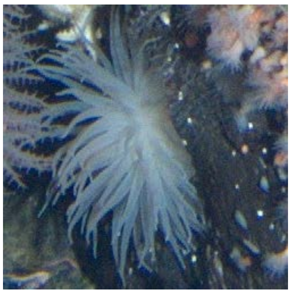

Zoantharia

Epizoanthus fatuus

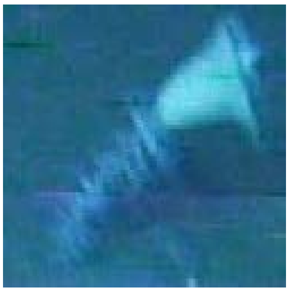

Epizoanthus sp.

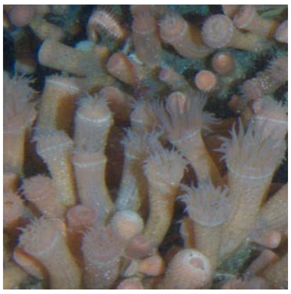

Zoantharia gen. sp.

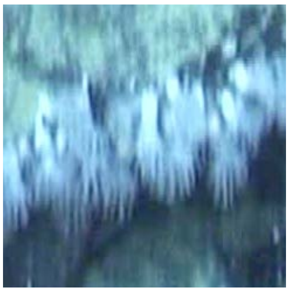

Scleractinia

Caryophylliidae gen. sp. 1

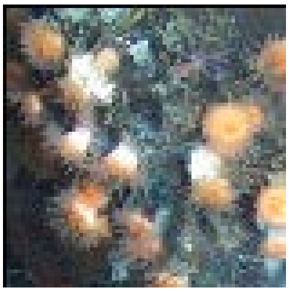

Caryophylliidae gen. sp. 2

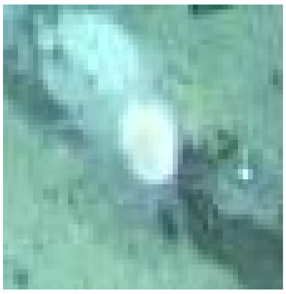

Corallimorpharia

Corallimorphus pilatus *Corallimorphus* sp. Corallimorphidae gen. sp

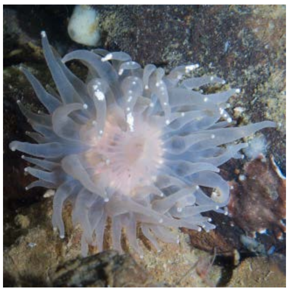
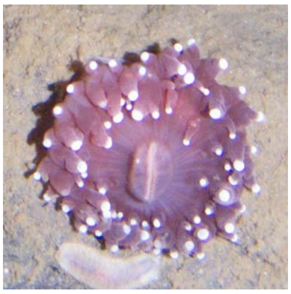
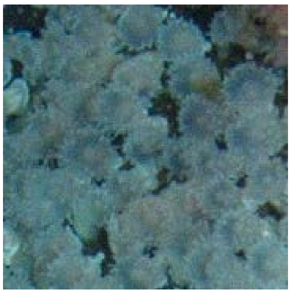

Antipatharia

Alternatipathes sp. Antipatharia gen. sp. Schizopathidae gen. sp. Antipatharia gen. sp.

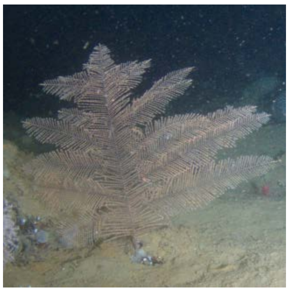
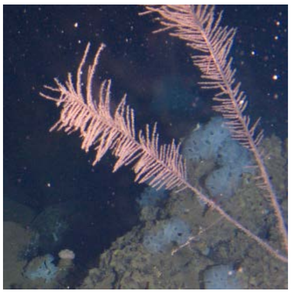
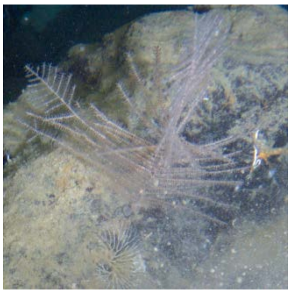
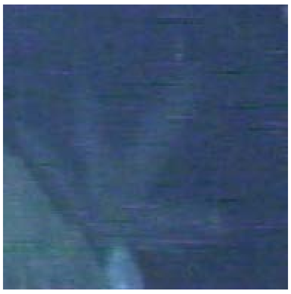

Polychaeta

Echiura gen. sp.

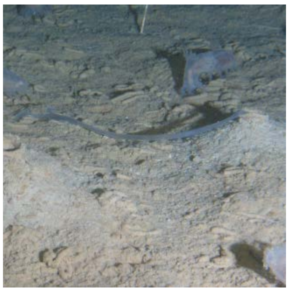

Mollusca

Polyplacophora

Polyplacophora gen. sp.1

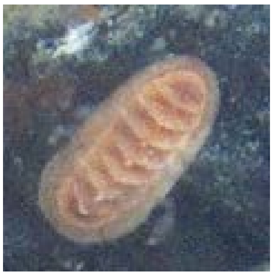

Polyplacophora gen. sp.2

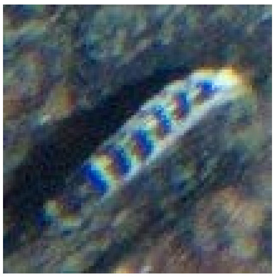

Polyplacophora gen. sp.3

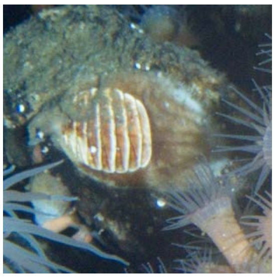

Gastropoda

Gastropoda gen. sp.1

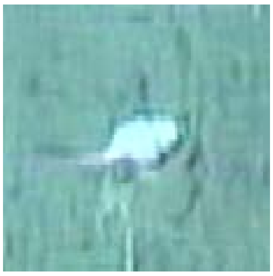

Gastropoda gen. sp.2

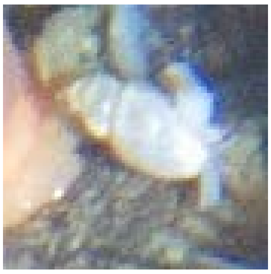

Gastropoda gen. sp.3

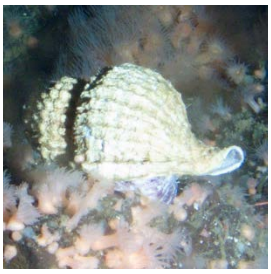

Tritonia cf. *diomedea*

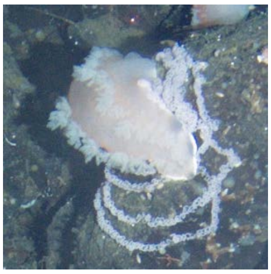

Cephalopoda

Muusoctopus profundorum

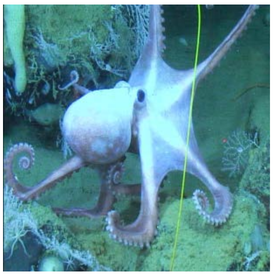

Cephalopoda gen. sp.2

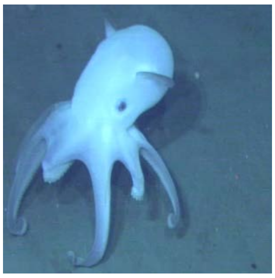

Arthropoda

Hexanauplia

Scalpellidae gen. sp.

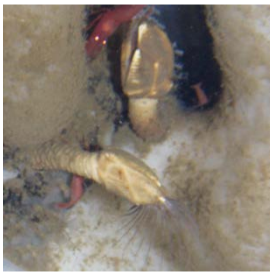

Malacostraca

Munidopsis gen. spp.

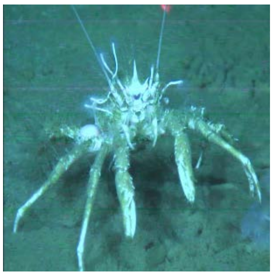

Galatheidae gen. spp.

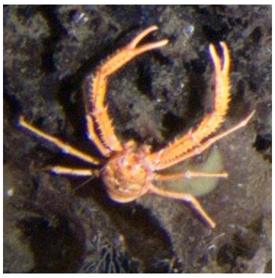

Lithodidae gen. spp.

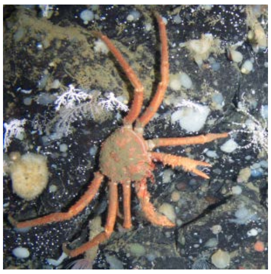

Chionoecetes sp.

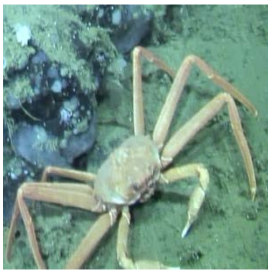

Oregoniidae gen. sp.

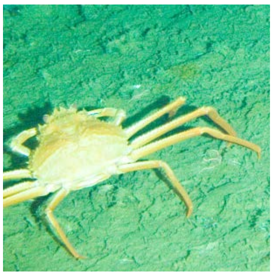

Crangonidae gen. sp.

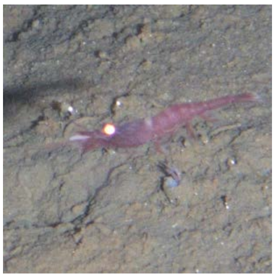

Pycnogonida

cf. *Colossendeis* sp.

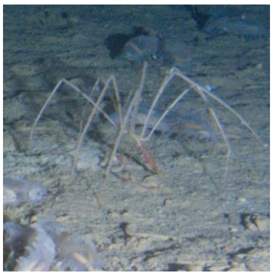

Echinodermata

Holothuroidea

Kolga kamchatica

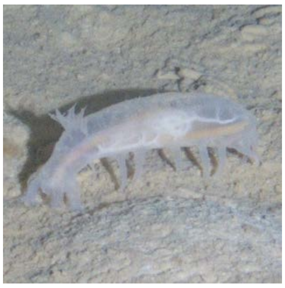

Scotoplanes kurilensis

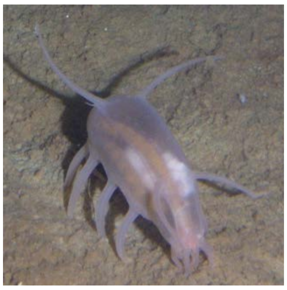

Zygothuria sp.1

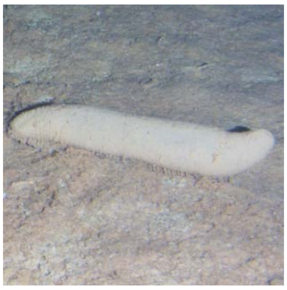

? *Zygothuria* sp.2

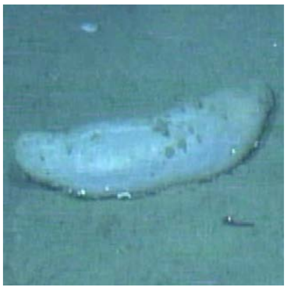

Psychropotes "*raripes*"

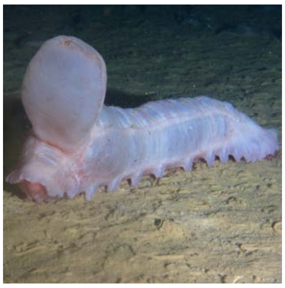

Psychropotes aff. *longicauda*

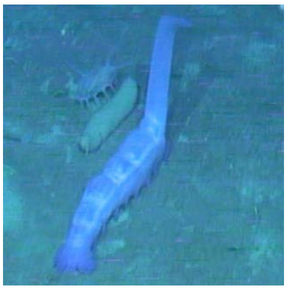

Paelopatides solea

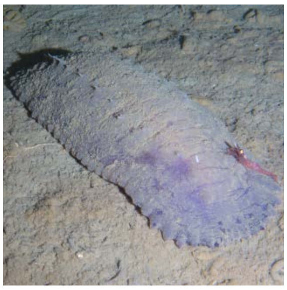

? Paelopatides sp.

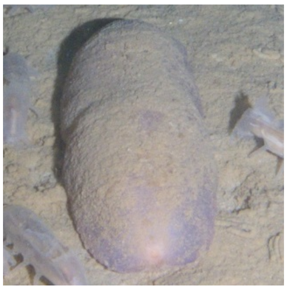

Synallactes chuni

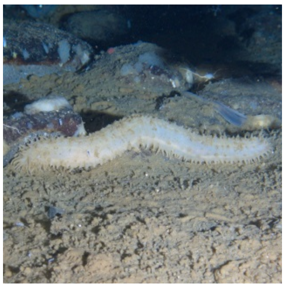

Synallactes sp. 1

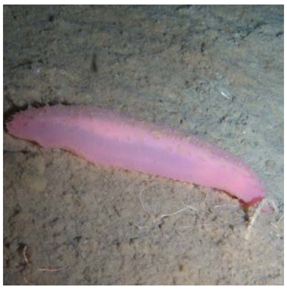

Synallactes sp. 2

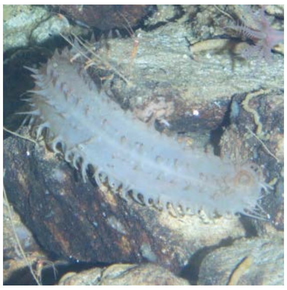

Synallactidae gen.sp.1

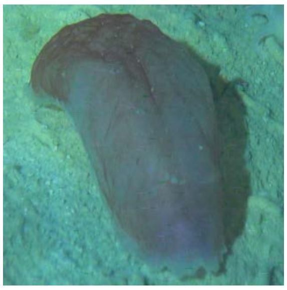

Synallactidae gen sp.2

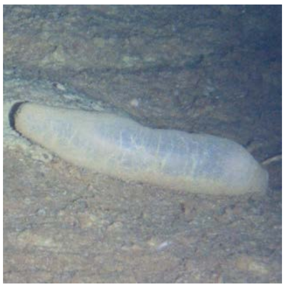

Pannychia aff. *moseleyi*

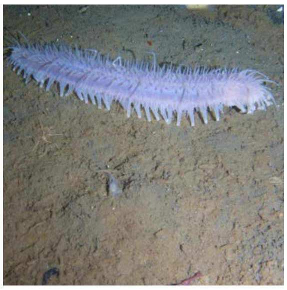

Psolus sp.

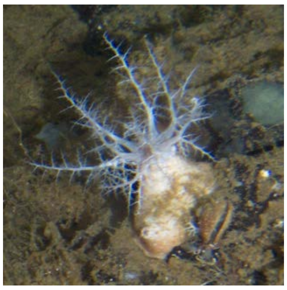

Abyssocucumis cf. *abyssorum*

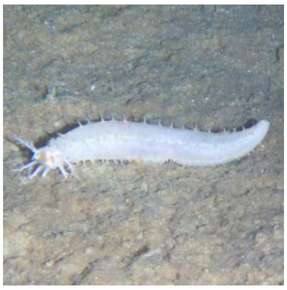

?Holothuroidea gen. sp.

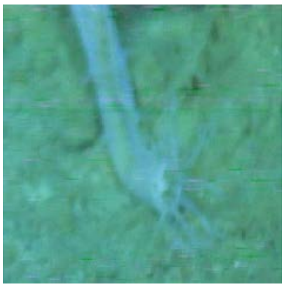

Ophiuroidea

Ophiacantha bathybia

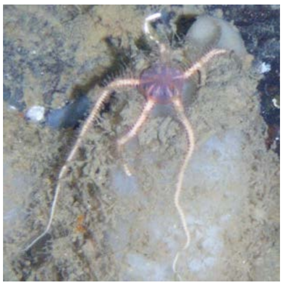

Ophiacantha cf. *enneactis*

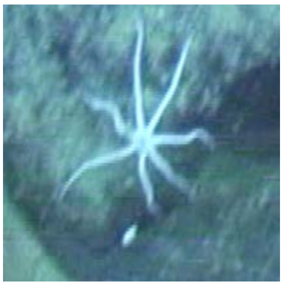

Ophiophthalmus sp.

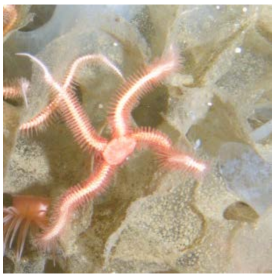

Ophiura bathybia

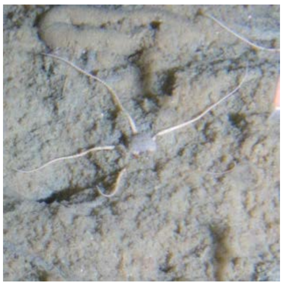

Ophiura sp.1

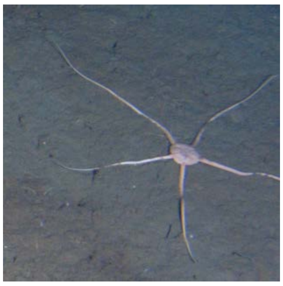

Ophiura sp.2

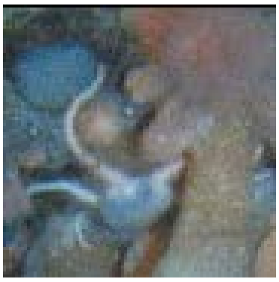

Astrochele laevis

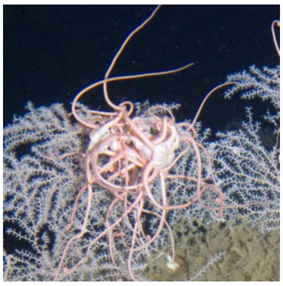

Ophiopholis cf. *japonica*

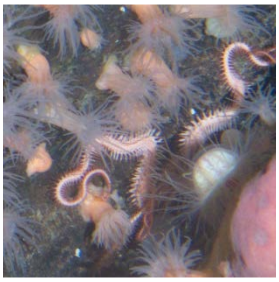

Asteroidea

Astrocles actinodetus

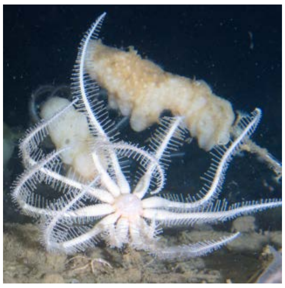

Freyella kurilokamchatica

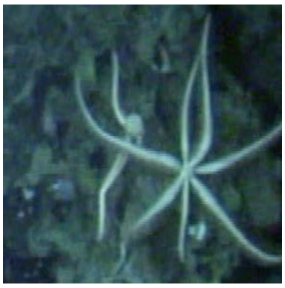

Freyellidae gen. sp.

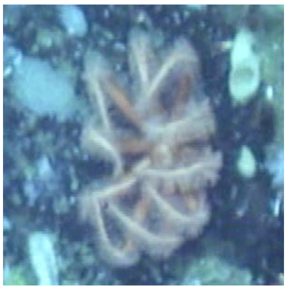

Hymenodiscus beringiana

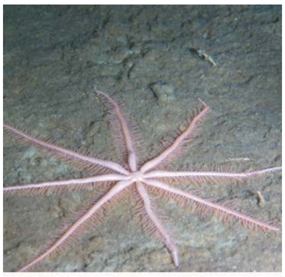

Brisingidae gen. sp.1

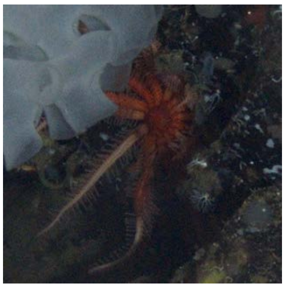

Brisingidae gen. sp.2

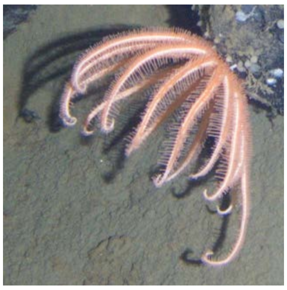

Brisingidae gen. sp. 3

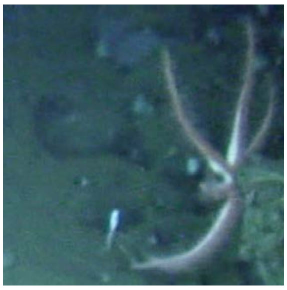

Hymenaster sp.

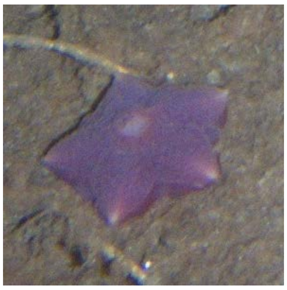

Pteraster sp. 1

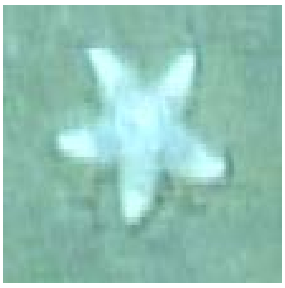

Pteraster sp. 2

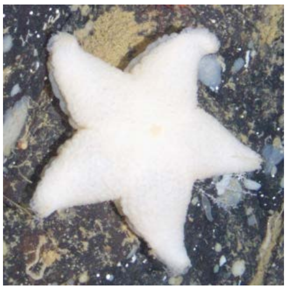

Crossaster papposus

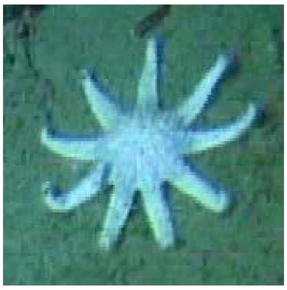

Crossaster sp.

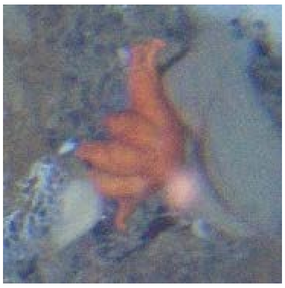

Lophaster sp.

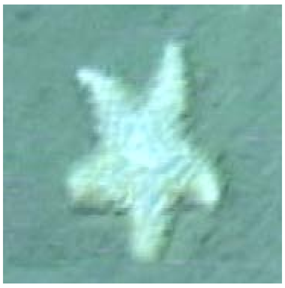

Solaster sp.

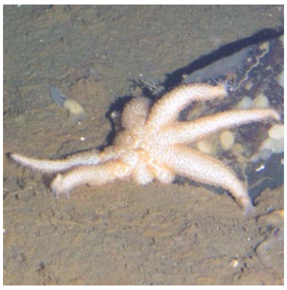

Nearchaster sp.

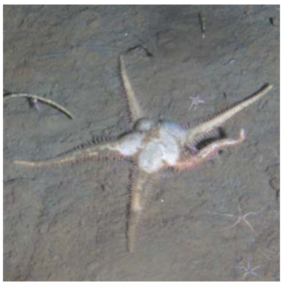

Hydrasterias sp.

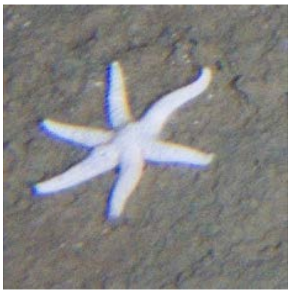

Hippasteria sp.

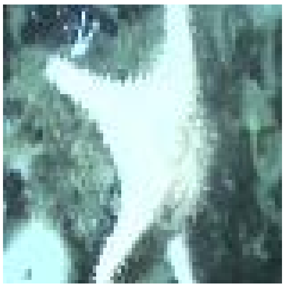

Goniasteridae gen.sp.1

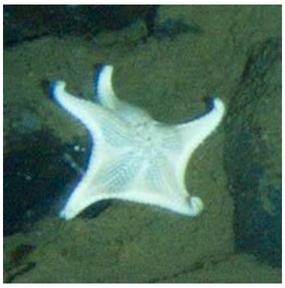

Goniasteridae gen.sp.2

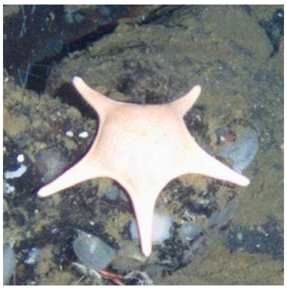

Goniasteridae gen.sp.3

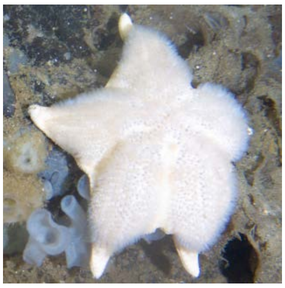

Henricia spp.

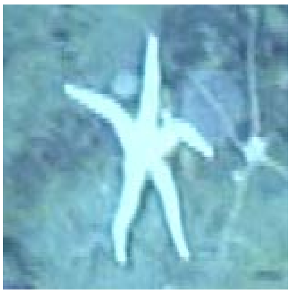

Hemichordata

Enteropneusta

Enteropneusta gen. sp.1

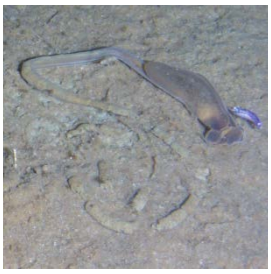

Torquaratoridae gen. sp.

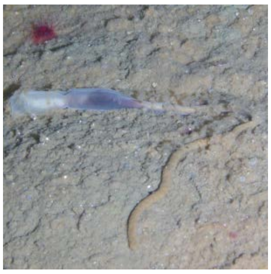

Chordata

Ascidiacea

Corynascidia sp.

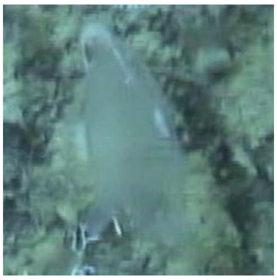

Ciona pomponiae

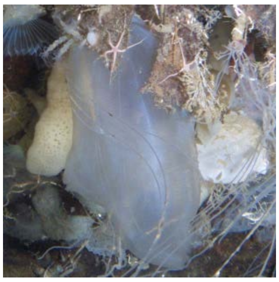

cf. *Bathypera ovoida*

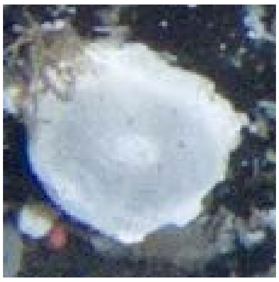

Boltenia echinata

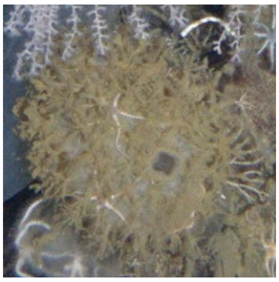

Megalodicopia hians

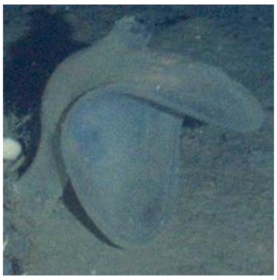

Cnemidocarpa ochotense

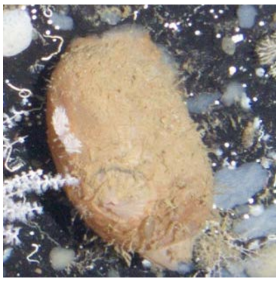

cf. Styelidae gen. sp.

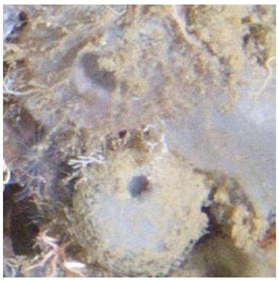

Aplousobranchia gen. sp.

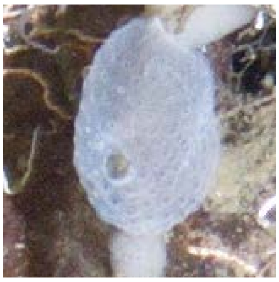

Supplement: Data S1 [file peerj-08-8628-s001.pdf]
